# Supplementary material for: Heparin-based hydrogel scaffolding alters the transcriptomic profile and increases the chemoresistance of MDA-MB-231 triple-negative breast cancer cells
Source: Biomater Sci. 2020 Feb 13;8(10):2786–96. doi: 10.1039/c9bm01481k (PMC7497406; doi:10.1039/c9bm01481k)
Supplement: Supplementary file 2 [file BM-008-C9BM01481K-s002.zip › Supplementary File 4/HP-B hydrogel + EGF (s)vControl/Pathways/my_analysis.Gsea.1545201170987/HALLMARK_MTORC1_SIGNALING.html]

Details for gene set HALLMARK\_MTORC1\_SIGNALING[GSEA]

|  || Dataset | expr.class.cls#HYDEGF\_versus\_CONTROL.class.cls#HYDEGF\_versus\_CONTROL\_repos |
| Phenotype | class.cls#HYDEGF\_versus\_CONTROL\_repos |
| Upregulated in class | CONTROL |
| GeneSet | HALLMARK\_MTORC1\_SIGNALING |
| Enrichment Score (ES) | -0.43830642 |
| Normalized Enrichment Score (NES) | -2.1026795 |
| Nominal p-value | 0.0 |
| FDR q-value | 0.0 |
| FWER p-Value | 0.0 |
Table: GSEA Results Summary

  

Fig 1: Enrichment plot: HALLMARK\_MTORC1\_SIGNALING      
 Profile of the Running ES Score & Positions of GeneSet Members on the Rank Ordered List

  

| PROBE | DESCRIPTION (from dataset) | GENE SYMBOL | GENE\_TITLE | RANK IN GENE LIST | RANK METRIC SCORE | RUNNING ES | CORE ENRICHMENT || 1 | DDX39A | na |  |  | 115 | 2.860 | 0.0109 | No |
| 2 | CORO1A | na |  |  | 222 | 2.536 | 0.0204 | No |
| 3 | SLC7A5 | na |  |  | 239 | 2.501 | 0.0343 | No |
| 4 | CCNF | na |  |  | 443 | 2.181 | 0.0366 | No |
| 5 | TOMM40 | na |  |  | 559 | 2.052 | 0.0427 | No |
| 6 | SLC1A5 | na |  |  | 679 | 1.922 | 0.0478 | No |
| 7 | MCM2 | na |  |  | 778 | 1.847 | 0.0536 | No |
| 8 | PPP1R15A | na |  |  | 1051 | 1.653 | 0.0491 | No |
| 9 | ASNS | na |  |  | 1455 | 1.448 | 0.0365 | No |
| 10 | SYTL2 | na |  |  | 1615 | 1.385 | 0.0364 | No |
| 11 | CDC25A | na |  |  | 1707 | 1.357 | 0.0396 | No |
| 12 | MAP2K3 | na |  |  | 2199 | 1.211 | 0.0210 | No |
| 13 | EBP | na |  |  | 2627 | 1.097 | 0.0050 | No |
| 14 | DDIT3 | na |  |  | 2748 | 1.066 | 0.0050 | No |
| 15 | PHGDH | na |  |  | 2820 | 1.048 | 0.0075 | No |
| 16 | MCM4 | na |  |  | 3125 | 0.976 | -0.0027 | No |
| 17 | CTH | na |  |  | 3158 | 0.969 | 0.0013 | No |
| 18 | RRP9 | na |  |  | 3207 | 0.957 | 0.0045 | No |
| 19 | SLC1A4 | na |  |  | 3358 | 0.931 | 0.0021 | No |
| 20 | IFRD1 | na |  |  | 3686 | 0.868 | -0.0099 | No |
| 21 | BUB1 | na |  |  | 4319 | 0.763 | -0.0387 | No |
| 22 | GOT1 | na |  |  | 4676 | 0.710 | -0.0532 | No |
| 23 | GGA2 | na |  |  | 4693 | 0.708 | -0.0498 | No |
| 24 | UNG | na |  |  | 4743 | 0.698 | -0.0483 | No |
| 25 | FDXR | na |  |  | 4868 | 0.674 | -0.0508 | No |
| 26 | DHCR24 | na |  |  | 4964 | 0.657 | -0.0519 | No |
| 27 | ABCF2 | na |  |  | 4986 | 0.654 | -0.0491 | No |
| 28 | PSAT1 | na |  |  | 5076 | 0.641 | -0.0500 | No |
| 29 | GSK3B | na |  |  | 5099 | 0.638 | -0.0474 | No |
| 30 | PIK3R3 | na |  |  | 5161 | 0.627 | -0.0469 | No |
| 31 | TRIB3 | na |  |  | 5408 | 0.590 | -0.0563 | No |
| 32 | IFI30 | na |  |  | 5554 | 0.570 | -0.0606 | No |
| 33 | HMBS | na |  |  | 5973 | 0.506 | -0.0795 | No |
| 34 | NMT1 | na |  |  | 6101 | 0.490 | -0.0833 | No |
| 35 | FGL2 | na |  |  | 6216 | 0.474 | -0.0865 | No |
| 36 | NUPR1 | na |  |  | 6404 | 0.449 | -0.0937 | No |
| 37 | PNP | na |  |  | 6968 | 0.376 | -0.1211 | No |
| 38 | ME1 | na |  |  | 7136 | 0.354 | -0.1278 | No |
| 39 | SLC37A4 | na |  |  | 7422 | 0.314 | -0.1409 | No |
| 40 | TUBA4A | na |  |  | 7430 | 0.313 | -0.1394 | No |
| 41 | PLK1 | na |  |  | 7470 | 0.309 | -0.1396 | No |
| 42 | TFRC | na |  |  | 7904 | 0.255 | -0.1609 | No |
| 43 | SDF2L1 | na |  |  | 7936 | 0.251 | -0.1610 | No |
| 44 | YKT6 | na |  |  | 8014 | 0.241 | -0.1637 | No |
| 45 | LDLR | na |  |  | 8020 | 0.241 | -0.1625 | No |
| 46 | TUBG1 | na |  |  | 8505 | 0.186 | -0.1868 | No |
| 47 | MTHFD2L | na |  |  | 8512 | 0.185 | -0.1861 | No |
| 48 | ITGB2 | na |  |  | 8519 | 0.184 | -0.1853 | No |
| 49 | RPA1 | na |  |  | 8622 | 0.168 | -0.1897 | No |
| 50 | SLC7A11 | na |  |  | 8638 | 0.165 | -0.1895 | No |
| 51 | MTHFD2 | na |  |  | 8784 | 0.152 | -0.1962 | No |
| 52 | GCLC | na |  |  | 8925 | 0.131 | -0.2028 | No |
| 53 | PNO1 | na |  |  | 8957 | 0.125 | -0.2037 | No |
| 54 | QDPR | na |  |  | 9298 | 0.089 | -0.2210 | No |
| 55 | ATP2A2 | na |  |  | 9352 | 0.081 | -0.2233 | No |
| 56 | NFKBIB | na |  |  | 9368 | 0.079 | -0.2236 | No |
| 57 | NUP205 | na |  |  | 9529 | 0.071 | -0.2316 | No |
| 58 | ACACA | na |  |  | 9794 | 0.037 | -0.2453 | No |
| 59 | RRM2 | na |  |  | 9839 | 0.030 | -0.2474 | No |
| 60 | G6PD | na |  |  | 10053 | 0.012 | -0.2586 | No |
| 61 | PFKL | na |  |  | 10128 | 0.001 | -0.2625 | No |
| 62 | FAM129A | na |  |  | 10690 | -0.064 | -0.2916 | No |
| 63 | SRD5A1 | na |  |  | 10696 | -0.064 | -0.2915 | No |
| 64 | COPS5 | na |  |  | 10746 | -0.072 | -0.2936 | No |
| 65 | SHMT2 | na |  |  | 10804 | -0.078 | -0.2962 | No |
| 66 | PDAP1 | na |  |  | 10816 | -0.080 | -0.2963 | No |
| 67 | SORD | na |  |  | 10833 | -0.083 | -0.2966 | No |
| 68 | SLC6A6 | na |  |  | 10932 | -0.099 | -0.3012 | No |
| 69 | TCEA1 | na |  |  | 11214 | -0.125 | -0.3152 | No |
| 70 | PSMG1 | na |  |  | 11242 | -0.129 | -0.3159 | No |
| 71 | AURKA | na |  |  | 11282 | -0.134 | -0.3171 | No |
| 72 | ARPC5L | na |  |  | 11479 | -0.160 | -0.3265 | No |
| 73 | PSPH | na |  |  | 11605 | -0.176 | -0.3320 | No |
| 74 | DHFR | na |  |  | 11640 | -0.180 | -0.3328 | No |
| 75 | TXNRD1 | na |  |  | 11750 | -0.196 | -0.3373 | No |
| 76 | CACYBP | na |  |  | 11818 | -0.206 | -0.3396 | No |
| 77 | CYP51A1 | na |  |  | 11845 | -0.211 | -0.3398 | No |
| 78 | HMGCS1 | na |  |  | 11862 | -0.213 | -0.3393 | No |
| 79 | ACLY | na |  |  | 12160 | -0.238 | -0.3535 | No |
| 80 | PSME3 | na |  |  | 12207 | -0.245 | -0.3545 | No |
| 81 | PSMC6 | na |  |  | 12333 | -0.266 | -0.3595 | No |
| 82 | BCAT1 | na |  |  | 12337 | -0.267 | -0.3581 | No |
| 83 | ADD3 | na |  |  | 12405 | -0.273 | -0.3600 | No |
| 84 | TMEM97 | na |  |  | 12724 | -0.318 | -0.3748 | No |
| 85 | IMMT | na |  |  | 12982 | -0.338 | -0.3864 | No |
| 86 | POLR3G | na |  |  | 13046 | -0.349 | -0.3876 | No |
| 87 | SLC2A1 | na |  |  | 13117 | -0.356 | -0.3892 | No |
| 88 | HSPA9 | na |  |  | 13160 | -0.362 | -0.3892 | No |
| 89 | GTF2H1 | na |  |  | 13241 | -0.376 | -0.3912 | No |
| 90 | UCHL5 | na |  |  | 13311 | -0.388 | -0.3926 | No |
| 91 | TM7SF2 | na |  |  | 13337 | -0.393 | -0.3915 | No |
| 92 | MLLT11 | na |  |  | 13372 | -0.398 | -0.3910 | No |
| 93 | PSMC4 | na |  |  | 13485 | -0.411 | -0.3944 | No |
| 94 | GPI | na |  |  | 13512 | -0.415 | -0.3933 | No |
| 95 | GMPS | na |  |  | 13591 | -0.423 | -0.3949 | No |
| 96 | GLA | na |  |  | 13717 | -0.444 | -0.3989 | No |
| 97 | EPRS | na |  |  | 13907 | -0.472 | -0.4060 | No |
| 98 | LGMN | na |  |  | 13973 | -0.484 | -0.4066 | No |
| 99 | SQSTM1 | na |  |  | 13980 | -0.486 | -0.4040 | No |
| 100 | BTG2 | na |  |  | 14115 | -0.493 | -0.4081 | No |
| 101 | HMGCR | na |  |  | 14351 | -0.528 | -0.4174 | No |
| 102 | HSPD1 | na |  |  | 14479 | -0.552 | -0.4208 | No |
| 103 | PDK1 | na |  |  | 14692 | -0.588 | -0.4284 | No |
| 104 | TBK1 | na |  |  | 14771 | -0.599 | -0.4290 | No |
| 105 | ETF1 | na |  |  | 14811 | -0.605 | -0.4274 | No |
| 106 | CDKN1A | na |  |  | 14882 | -0.616 | -0.4275 | No |
| 107 | SERPINH1 | na |  |  | 15083 | -0.648 | -0.4342 | No |
| 108 | NFYC | na |  |  | 15163 | -0.662 | -0.4344 | Yes |
| 109 | HSPA4 | na |  |  | 15199 | -0.669 | -0.4323 | Yes |
| 110 | SLC9A3R1 | na |  |  | 15259 | -0.680 | -0.4313 | Yes |
| 111 | CCT6A | na |  |  | 15268 | -0.683 | -0.4277 | Yes |
| 112 | ATP5G1 | na |  |  | 15307 | -0.688 | -0.4256 | Yes |
| 113 | HSPE1 | na |  |  | 15347 | -0.696 | -0.4235 | Yes |
| 114 | RPN1 | na |  |  | 15354 | -0.697 | -0.4197 | Yes |
| 115 | PSMD13 | na |  |  | 15416 | -0.704 | -0.4188 | Yes |
| 116 | PSMA3 | na |  |  | 15431 | -0.706 | -0.4153 | Yes |
| 117 | ADIPOR2 | na |  |  | 15521 | -0.725 | -0.4157 | Yes |
| 118 | PSMD14 | na |  |  | 15549 | -0.732 | -0.4128 | Yes |
| 119 | SEC11A | na |  |  | 15723 | -0.769 | -0.4173 | Yes |
| 120 | SERP1 | na |  |  | 15766 | -0.778 | -0.4149 | Yes |
| 121 | CTSC | na |  |  | 15844 | -0.790 | -0.4143 | Yes |
| 122 | NFIL3 | na |  |  | 15878 | -0.797 | -0.4113 | Yes |
| 123 | M6PR | na |  |  | 15895 | -0.801 | -0.4074 | Yes |
| 124 | EEF1E1 | na |  |  | 15903 | -0.802 | -0.4030 | Yes |
| 125 | HK2 | na |  |  | 15926 | -0.806 | -0.3994 | Yes |
| 126 | XBP1 | na |  |  | 16049 | -0.833 | -0.4009 | Yes |
| 127 | FADS2 | na |  |  | 16193 | -0.868 | -0.4032 | Yes |
| 128 | DDIT4 | na |  |  | 16203 | -0.871 | -0.3985 | Yes |
| 129 | STIP1 | na |  |  | 16399 | -0.923 | -0.4033 | Yes |
| 130 | PSMB5 | na |  |  | 16435 | -0.929 | -0.3997 | Yes |
| 131 | DHCR7 | na |  |  | 16459 | -0.938 | -0.3953 | Yes |
| 132 | UBE2D3 | na |  |  | 16494 | -0.946 | -0.3915 | Yes |
| 133 | ALDOA | na |  |  | 16587 | -0.967 | -0.3906 | Yes |
| 134 | GSR | na |  |  | 16589 | -0.967 | -0.3849 | Yes |
| 135 | ATP6V1D | na |  |  | 16591 | -0.967 | -0.3792 | Yes |
| 136 | SQLE | na |  |  | 16592 | -0.967 | -0.3735 | Yes |
| 137 | TES | na |  |  | 16614 | -0.974 | -0.3688 | Yes |
| 138 | ELOVL6 | na |  |  | 16631 | -0.979 | -0.3639 | Yes |
| 139 | UFM1 | na |  |  | 16671 | -0.992 | -0.3601 | Yes |
| 140 | PSMC2 | na |  |  | 16681 | -0.995 | -0.3546 | Yes |
| 141 | FKBP2 | na |  |  | 16776 | -1.018 | -0.3535 | Yes |
| 142 | FADS1 | na |  |  | 16789 | -1.024 | -0.3481 | Yes |
| 143 | BHLHE40 | na |  |  | 16798 | -1.027 | -0.3424 | Yes |
| 144 | NUFIP1 | na |  |  | 16821 | -1.034 | -0.3375 | Yes |
| 145 | RIT1 | na |  |  | 16839 | -1.039 | -0.3322 | Yes |
| 146 | PSMD12 | na |  |  | 16859 | -1.044 | -0.3270 | Yes |
| 147 | PSMA4 | na |  |  | 16892 | -1.051 | -0.3225 | Yes |
| 148 | CYB5B | na |  |  | 16935 | -1.066 | -0.3184 | Yes |
| 149 | STARD4 | na |  |  | 16981 | -1.083 | -0.3143 | Yes |
| 150 | EIF2S2 | na |  |  | 17005 | -1.091 | -0.3090 | Yes |
| 151 | CXCR4 | na |  |  | 17068 | -1.103 | -0.3058 | Yes |
| 152 | EDEM1 | na |  |  | 17083 | -1.110 | -0.2999 | Yes |
| 153 | INSIG1 | na |  |  | 17226 | -1.153 | -0.3006 | Yes |
| 154 | CFP | na |  |  | 17242 | -1.156 | -0.2945 | Yes |
| 155 | PITPNB | na |  |  | 17382 | -1.210 | -0.2946 | Yes |
| 156 | WARS | na |  |  | 17398 | -1.213 | -0.2882 | Yes |
| 157 | ELOVL5 | na |  |  | 17447 | -1.235 | -0.2834 | Yes |
| 158 | PLOD2 | na |  |  | 17461 | -1.240 | -0.2768 | Yes |
| 159 | PGM1 | na |  |  | 17488 | -1.254 | -0.2707 | Yes |
| 160 | ACTR2 | na |  |  | 17574 | -1.284 | -0.2676 | Yes |
| 161 | CALR | na |  |  | 17688 | -1.326 | -0.2656 | Yes |
| 162 | TPI1 | na |  |  | 17749 | -1.359 | -0.2607 | Yes |
| 163 | HSPA5 | na |  |  | 17766 | -1.367 | -0.2535 | Yes |
| 164 | VLDLR | na |  |  | 17779 | -1.371 | -0.2460 | Yes |
| 165 | CCNG1 | na |  |  | 17820 | -1.396 | -0.2398 | Yes |
| 166 | ACSL3 | na |  |  | 17998 | -1.484 | -0.2403 | Yes |
| 167 | PRDX1 | na |  |  | 18026 | -1.499 | -0.2329 | Yes |
| 168 | PPA1 | na |  |  | 18039 | -1.507 | -0.2246 | Yes |
| 169 | IDI1 | na |  |  | 18098 | -1.547 | -0.2184 | Yes |
| 170 | USO1 | na |  |  | 18109 | -1.553 | -0.2098 | Yes |
| 171 | ACTR3 | na |  |  | 18277 | -1.659 | -0.2087 | Yes |
| 172 | HPRT1 | na |  |  | 18443 | -1.794 | -0.2068 | Yes |
| 173 | LTA4H | na |  |  | 18508 | -1.843 | -0.1992 | Yes |
| 174 | SKAP2 | na |  |  | 18509 | -1.844 | -0.1883 | Yes |
| 175 | P4HA1 | na |  |  | 18626 | -1.958 | -0.1828 | Yes |
| 176 | GLRX | na |  |  | 18639 | -1.972 | -0.1717 | Yes |
| 177 | RAB1A | na |  |  | 18658 | -1.997 | -0.1608 | Yes |
| 178 | IDH1 | na |  |  | 18690 | -2.035 | -0.1504 | Yes |
| 179 | SSR1 | na |  |  | 18693 | -2.038 | -0.1384 | Yes |
| 180 | RDH11 | na |  |  | 18715 | -2.072 | -0.1272 | Yes |
| 181 | AK4 | na |  |  | 18825 | -2.239 | -0.1197 | Yes |
| 182 | GBE1 | na |  |  | 18855 | -2.282 | -0.1077 | Yes |
| 183 | SCD | na |  |  | 18875 | -2.317 | -0.0949 | Yes |
| 184 | STC1 | na |  |  | 18901 | -2.351 | -0.0823 | Yes |
| 185 | NAMPT | na |  |  | 18985 | -2.547 | -0.0716 | Yes |
| 186 | HSP90B1 | na |  |  | 19003 | -2.588 | -0.0571 | Yes |
| 187 | CD9 | na |  |  | 19017 | -2.631 | -0.0422 | Yes |
| 188 | SLC2A3 | na |  |  | 19022 | -2.648 | -0.0267 | Yes |
| 189 | EGLN3 | na |  |  | 19047 | -2.739 | -0.0118 | Yes |
| 190 | PGK1 | na |  |  | 19154 | -3.315 | 0.0023 | Yes |
Table: GSEA details [plain text format]

  

Fig 2: HALLMARK\_MTORC1\_SIGNALING      
 Blue-Pink O' Gram in the Space of the Analyzed GeneSet

  

Fig 3: HALLMARK\_MTORC1\_SIGNALING: Random ES distribution      
 Gene set null distribution of ES for **HALLMARK\_MTORC1\_SIGNALING**
